# Implications of neuroendocrine tumor and diabetes mellitus on patient outcomes and care: a matched case–control study

**DOI:** 10.2144/fsoa-2020-0190

**Published:** 2021-02-15

**Authors:** Yael N Kusne, Heidi E Kosiorek, Matthew R Buras, Patricia M Verona, Kyle E Coppola, Kelley A Rone, Curtiss B Cook, Nina J Karlin

**Affiliations:** 1 Department of Internal Medicine, Mayo Clinic, Scottsdale 85259, Arizona; 2 Biostatistics, Mayo Clinic, Scottsdale 85259, Arizona; 3 Enterprise Technology Services, Mayo Clinic, Scottsdale 85259, Arizona; 4 Mayo Clinic Cancer Center, Mayo Clinic, Scottsdale 85259, Arizona; 5 Division of Endocrinology, Mayo Clinic, Scottsdale 85259, Arizona; 6 Division of Hematology & Medical Oncology, Mayo Clinic Hospital, Phoenix 85054, Arizona

**Keywords:** cancer, endocrinology, prognostics

## Abstract

**Aim:**

We aimed to determine the impact of diabetes mellitus (DM) on survival of patients with neuroendocrine tumors (NETs) and of NETs on glycemic control.

**Patients & methods:**

Patients with newly diagnosed NETs with/without DM were matched 1:1 by age, sex and diagnosis year (2005–2017), and survival compared (Kaplan–Meier and Cox proportional hazards). Mixed models compared hemoglobin A_1c_ (HbA_1c_) and glucose during the year after cancer diagnosis.

**Results:**

Three-year overall survival was 72% (95% CI: 60–86%) for DM patients versus 80% (95% CI: 70–92%) for non-DM patients (p = 0.82). Hazard ratio was 1.33 (95% CI: 0.56–3.16; p = 0.51); mean DM HbA_1c_, 7.3%.

**Conclusion:**

DM did not adversely affect survival of patients with NET. NET and its treatment did not affect glycemic control.

## Lay abstract

The aim of this study was to evaluate the effect of diabetes mellitus (DM) on survival of patients with neuroendocrine tumor (NET) and to determine whether NET affected glycemic control. From an institutional cancer registry, 118 patients with NET were identified and grouped by DM (n = 59) or no DM (n = 59). The two groups were matched by age, sex and year of NET diagnosis. DM did not decrease survival, and NET did not significantly affect glycemic control in patients with DM.

Neuroendocrine tumors (NETs) comprise a heterogeneous tumor group that can be classified via embryologic origin, anatomic site of occurrence and/or secretion of peptides or neuroamines. The locations of these tumors vary, but most originate within the gastroenteropancreatic tract. There are both sporadic and hereditary NETs, but most cases are sporadic (90%) [1]. Inherited NETs are associated with numerous syndromes including multiple endocrine neoplasia type 1 (MEN-1 syndrome) or type 2 (MEN-2 syndrome) and von Hippel-Lindau syndrome, among others [2]. Among NETs identified in the foregut (pancreas, stomach and duodenum), estimates range from 2 to 40% for MEN-1 mutations, and the resulting cases have no genetic cause identified. The incidence of NETs has been increasing in all anatomic locations. In 1973, the overall incidence was 1.09 per 100,000 population, and in 2012 it increased to 6.98 per 100,000 population [3]. The median overall survival for patients with NET has been estimated at 9.3 years, with significantly increased overall survival for localized tumors in all anatomic locations compared with metastatic NETs [3,4]. Anatomic location also significantly affects prognosis. The 5-year survival rate for enteric NETs is estimated to be 67%, whereas for pancreatic NETs, survival rates are highly varied depending on type [4–7]. For example, of pancreatic NETs, 20–30% are insulinoma, which have a 5-year survival rate of 80–95%, whereas somatostatinomas (0–1%) have a 5-year survival rate of 20–40% [4,8].

Some types of NETs, such as insulinomas and glucagonomas, have been associated with abnormalities of glucose metabolism. For example, up to 83% of patients with glucagonomas may present with diabetes mellitus (DM) [9]. In a study of 21 patients with glucagonomas, 16 patients developed DM, and 75% of those required insulin therapy [10]. Additionally, several drugs used to treat NETs may potentially impact glycemic control. Somatostatin analogs, which are commonly used to treat NETs, may alter glycemic balance via inhibition of insulin and glucagon secretion [11,12]. Additionally, everolimus either alone or in combination with a somatostatin analog has been associated with hyperglycemia in various clinical trials [12]. Given the frequency of impaired glucose regulation, it has been recommended that patients with NETs with or without preexisting DM be monitored closely for alterations in blood glucose and hemoglobin A_1c_ (HbA_1c_) levels.

In many different cancer types, preexisting DM has been linked to worse all-cause mortality [13]. As with most cancers, data have also shown DM to be a risk factor for development of NET [14,15]; however, there are conflicting results from analysis of outcomes and survival in patients with DM and NETs. In epidemiologic analysis, patients with DM had a more advanced tumor stage at diagnosis compared with those without DM [16]. Another epidemiologic study failed to replicate this result [17]. In a retrospective analysis of 445 patients with pancreatic NETs, progression-free survival for patients with DM was significantly improved compared with that in non-DM patients, although this was thought to be related to metformin use [18]. Another study of 1535 patients with NETs did not find any change in mortality or survival rates in patients with or without DM who were being treated with radiopeptide therapy. Additionally, this study noted that radiopeptide therapy did not increase the risk of developing DM in patients with NETs [19]. The lack of survival benefit for DM patients was confirmed in a more recent study of 299 patients with pancreatic NETs in which DM was not found to influence prognosis [20]; however, DM patients had a higher likelihood of tumor metastases. In another recent study, patients with a pancreatic NET and preoperative hyperglycemia but without a preexisting diagnosis of DM (defined by a blood glucose level of 140–198 mg/dl 3 days before surgery and an HbA_1c_ level <6.5% 3 months before surgery) were shown to have a greater rate of metastatic disease and worse overall and recurrence-free survival than patients with preexisting DM and normal blood glucose levels [21].

Given the lack of consensus in published studies, the aims of this study were to examine the impact of preexisting DM on the outcomes of patients being treated for NET and to determine whether the treatment affects glycemic control in these patients.

## Patients & methods

### Case selection

After institutional review board approval was obtained, we selected NET cases from the institutional cancer registry. Patients with newly diagnosed NET were identified between 1 January 2005 and 31 December 2017. Patient data collected from the electronic health record included demographic information and co-morbid conditions, cancer location and date of diagnosis, histology if available, therapy, and survival. Cancer data were linked to the electronic health record to identify individuals with DM, as previously described [22–27]. For patients with DM, data collected included diabetic therapy, HbA_1c_, glucose levels and complications of DM before and after cancer diagnosis. Patients were then matched 1:1 by age, sex and year of cancer diagnosis.

### Statistical analyses

For all patients with NETs who were included, baseline demographic information was compared between those with and without DM. To determine the level of glycemic control in the study population, the percentage of patients with HbA_1c_ levels less than 7% was calculated. HbA_1c_ and glucose levels during the first year after NET diagnosis were analyzed via mixed models with fixed and random effects. The Kaplan–Meier method was used to estimate 3-year overall survival. Overall survival was defined as the time from NET diagnosis until death from any cause. Patients without a date of death documented in the electronic health record were considered censored at the last known follow-up date. To determine the effect of DM on overall survival and progression-free survival, Cox proportional hazards regression was used, with matched pairs as the strata variable. p-values less than 0.05 were considered statistically significant. SAS version 9.4 (SAS Institute Inc.) was used for statistical analysis.

## Results

### Patient characteristics

In total, 118 patients (59 matched pairs) were identified and included in the analyses. Demographic information for the included patients is described in Table 1. No differences were detected between groups for race, tumor location, histologic findings, pathologic findings or type of treatment. The mean patient age at diagnosis was 67 years, and 22% of patients had pancreatic NETs, 45% had nonpancreatic gastrointestinal NETs and 17% had lung/thymus NETs. Of the total patients, 41% had stage IV disease, and 70% had well-differentiated pathology. Mean standard deviation (SD) body mass index was significantly different between patients with and without DM (31.0 [7.9] vs 26.4 [5.3]; p = 0.01). Glucose levels differed significantly between patients with DM (159.1 [43.5] mg/dl) and those without DM (117 [31.5] mg/dl; p < 0.001).

**Table 1.** Demographic characteristics related to neuroendocrine cancer and diabetes mellitus status.

	Diabetes mellitus, n (%)^†^			p-value
	No (n = 59)	Yes (n = 59)	Total (n = 118)	
Age at cancer diagnosis (years), mean (SD)	62.4 (11.9)	62.6 (12.2)	62.5 (12.0)	Matched
Race				0.46
White	43 (72.9)	48 (81.4)	91 (77.1)	
Non-White	7 (11.9)	2 (3.4)	9 (7.6)	
Unknown	9 (15.2)	9 (15.2)	18 (15.2)	
Ethnicity				0.48
Hispanic	5 (8.5)	2 (3.4)	7 (5.9)	
Non-Hispanic	44 (74.6)	48 (81.4)	92 (78.0)	
Unknown	10 (16.9)	9 (15.2)	19 (16.1)	
Anatomic location				0.17
Pancreas	9 (16.1)	16 (27.1)	25 (21.7)	
GI (not pancreas)	21 (37.5)	31 (52.5)	52 (45.2)	
Lung/thymus	14 (25.0)	6 (10.2)	20 (17.4)	
Unknown primary	4 (7.1)	4 (6.8)	8 (7.0)	
Other	8 (14.3)	2 (3.4)	10 (8.7)	
Missing data	3	0	3	
Tumor stage				0.20
I	14 (27.5)	22 (40.0)	36 (34.0)	
II	4 (7.8)	6 (10.9)	10 (9.4)	
III	7 (13.7)	10 (18.2)	17 (16.0)	
IV	26 (51.0)	17 (30.9)	43 (40.6)	
Missing	8	4	12	
BMI, mean (SD)	26.4 (5.3)	31.0 (7.9)	28.8 (7.1)	0.01
Smoking status at cancer diagnosis				0.24
Never	33 (57.9)	27 (45.8)	60 (51.7)	
Former	17 (29.8)	19 (32.2)	36 (31.0)	
Current	4 (7.0)	3 (5.1)	7 (6.0)	
Unknown	3 (5.3)	10 (16.9)	13 (11.2)	
Missing	2	0	2	
Surgery, n (%)				0.23
No	22 (41.5)	17 (28.8)	39 (34.8)	
Yes	31 (58.5)	42 (71.2)	73 (65.2)	
Missing	6	0	6	

† n (%) unless indicated otherwise.

GI: Gastrointestinal; SD: Standard deviation.

There were no differences in cancer treatment between patients with and without DM. There was no significant difference for patients who underwent surgery between groups (p = 0.23).

Most patients with DM were taking oral medications. For the majority of the patients, diabetes therapy did not change over the course of the year. For those whose therapy changed, six patients started insulin, and one switched to diet. Details can be found in Table 2.

**Table 2.** Treatment of diabetes mellitus.

Therapy	n (%) (n = 59)
DM therapy	
Diet	10 (22.7)
Oral	19 (43.2)
Insulin	11 (25.0)
Oral + insulin	3 (6.8)
Other	1 (2.3)
Missing	15
Change in method of DM therapy within 1 year after cancer diagnosis	
Yes	7 (13.7)
No	24 (47.1)
Unknown	20 (39.2)
Missing	8
Alternate method of DM therapy within 1 year after cancer diagnosis	
Diet	1 (14.3)
Insulin	6 (85.7)
Missing	52
Insulin use at time of cancer diagnosis	
Yes	13 (25.5)
No	31 (60.8)
Unknown	7 (13.7)
Missing	8
Insulin use within 1 year after cancer diagnosis	
Yes	18 (35.3)
No	18 (35.3)
Unknown	15 (29.4)
Missing	8
History of DM complications (before cancer diagnosis)	
Yes	1 (2.5)
No	2 (5.0)
Unknown	37 (92.5)
Missing	19
DM complications within 1 year after cancer diagnosis	
Yes	1 (2.0)
No	2 (3.9)
Unknown	48 (94.1)
Missing	8

DM: Diabetes mellitus.

### Cancer effect on glycemic control

**Figure 1.**
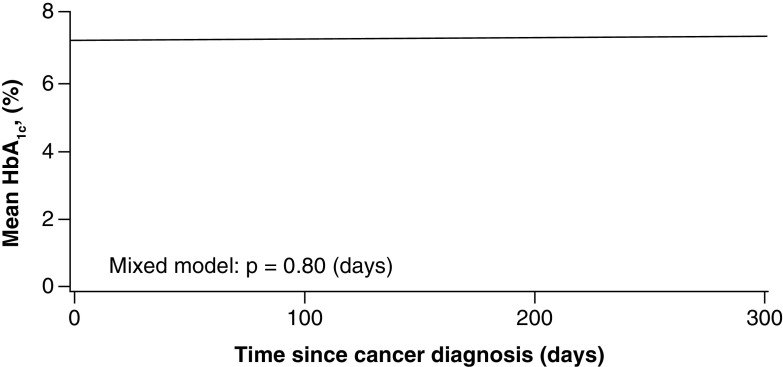
Mean HbA_1c_ level during the year after cancer diagnosis for patients with diabetes mellitus. The mean HbA_1c_ level did not change significantly during the course of cancer treatment. HbA_1c_ indicates hemoglobin A_1c_.

Among those with DM, the mean HbA_1c_ level during the year following cancer diagnosis was 7.3% (Figure 1) and did not change significantly during the course of cancer treatment. At the time of cancer diagnosis, 25% of patients with DM were using insulin; 1 year after cancer diagnosis, this increased to 35%. No significant increases in DM complications occurred 1 year after cancer diagnosis.

**Figure 2.**
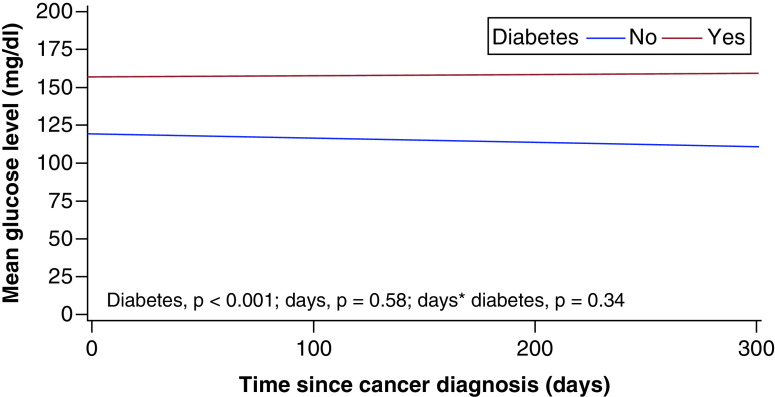
Mean (SD) glucose level for patients with and without diabetes mellitus. No significant changes occurred in glycemic control in either group over 12 months (p = 0.34, interaction effect; p = 0.58, time effect). *Main effects and interactions.

The mean (SD) glucose level was significantly different for patients with DM (159.1 [43.5] mg/dl) than for those without DM (117 [31.5] mg/dl; p < 0.001) (Figure 2); however, no significant changes occurred in glycemic control in either group over 12 months (p = 0.34 for interaction effect and p = 0.58 for time effect).

### DM effect on survival

**Figure 3.**
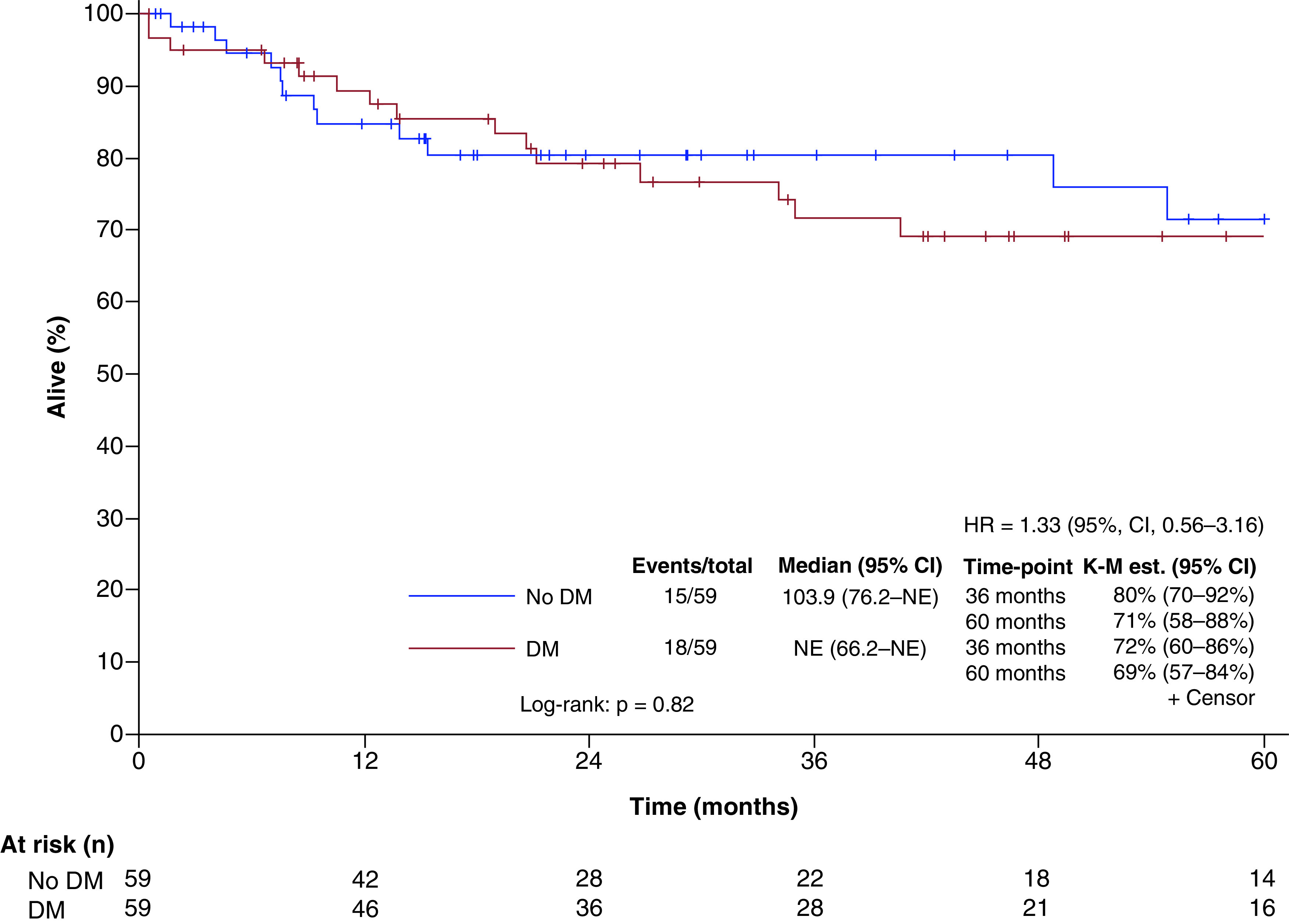
Overall survival for patients with and without diabetes mellitus (K–M method). DM: Diabetes mellitus; HR: Hazard ratio; K–M: Kaplan–Meier; NE: Not evaluable.

Median (range) follow-up was 32.8 (2.4–165.4) months (Figure 3). Three-year survival was estimated at 72% (95% CI: 60–86%) for patients with DM versus 80% (95% CI: 70–92%) for patients without DM (Kaplan–Meier method; p = 0.82, log-rank test). Five-year overall survival was 69% (95% CI: 57–84%) for patients with DM and 71% (95% CI: 58–88%) for patients without DM. The hazard ratio (stratification for matched pairs) was 1.33 (95% CI: 0.56–3.16; p = 0.51).

## Discussion

Existing evidence has shown DM to be a risk factor for the development of various malignancies including NETs [14,15,28,29]. A systematic review and meta-analysis showed that DM was a significant risk factor in development of pancreatic and gastric NETs among all case–control studies examined, and the overall risk was greater in patients with DM who were receiving insulin [15,30]. Interestingly, this association was not shown for NETs of the small intestine, rectum or lung [15,16,30,31]. Although NETs are considered rare, the incidence is increasing and DM, which is also increasing in incidence worldwide, is 1 of the most common co-morbid conditions among patients with NETs [32].

Despite this, the impact of DM on the prognosis of NETs has not been well studied, and the published studies have provided conflicting evidence. For example, Capurso *et al.* [16] reported a history of DM to be associated with metastatic disease at diagnosis, but a more recent study by Ben *et al.* [17] did not find such an association. Additionally, Sandini *et al.* [21] recently showed that preoperative hyperglycemia, but not preexisting DM, was associated with increased risk of metastatic disease and larger tumor size. Should patients with DM present with higher-grade disease and have increased risk of metastases, we would expect studies to show that the DM patients had poorer outcomes than non-DM patients. However, published studies have not found an association between DM and worse outcomes [19,20]. This is intriguing and certainly requires further study.

Our study had a case–control design, wherein patients were matched 1:1 according to age, sex and year of NET diagnosis, and we did not find an effect of DM on survival or any effect of NET on glycemic control in patients with preexisting DM. We examined data from 118 patients diagnosed with various NETs, which to our knowledge is the largest case–control evaluation to date. With the increasing incidence of DM, our results should be reassuring to oncologists and endocrinologists who treat patients with multiple co-morbid conditions. However, given the paucity of clinical data, close monitoring of blood glucose and HbA_1c_ levels in patients diagnosed with secretory NETs may be appropriate.

Our previously published case-matched studies for colorectal, pancreatic, lung and prostate cancers did not show an interaction between DM and cancer survival [22–25]. The only exception was for patients with gastroesophageal cancers, where we did find that patients with preexisting DM had a higher risk of death than those without DM [26]. For patients with melanoma, our analyses revealed an unexpected increase in progression-free survival in the DM group compared with the non-DM group [27]. The reasons for these differences are not clear; however, further studies should continue to explore these associations, as worldwide incidence of DM continues to rise. Our previously published case-matched studies have also consistently shown that solid tumors do not negatively impact glycemic control.

Limitations of our study include its retrospective design and small-sample size. The study also had a mostly White population; therefore, results may not be applicable to patients of other racial/ethnic backgrounds. Because of the rarity of cases, all NET types were grouped together for analysis, which may have affected outcomes. Therefore, future studies should further be refined by NET type as well as Ki67 and tumor grade. In addition, data on management of DM were recorded for oral versus insulin therapy, but data for the specific use of metformin was not collected, which would be important to evaluate as studies have recently shown metformin to be a potential contributor to survival in patients with pancreatic NETs [18,33,34] through a mechanism related to dose-dependent suppression of cell proliferation [35,36].

## Conclusion

In this matched case–control study of patients with DM and NET, we did not find a significant interaction between DM and survival. This is relevant for clinicians who are treating increasing numbers of patients who have both DM and malignancies, given the rising incidence of DM throughout the world.

## Future perspective

Given the study findings, medical providers can be reassured that DM does not affect survival of patients with NETs and that neuroendocrine tumor does not negatively impact glycemic control in patients with DM.

**Summary points**

- The impact of neuroendocrine tumor on diabetes mellitus (DM) and the impact of DM on survival of patients with neuroendocrine tumor are unknown on an individual level.
- BMI was significantly different between patients with and without DM (31.0 [7.9] vs 26.4 [5.3]; p = 0.01).
- Among those with DM, mean hemoglobin A_1c_ was 7.3% during the year after cancer diagnosis.
- The mean (SD) glucose level was significantly different between patients with (159.1 [43.5] mg/dl) versus without DM (117 [31.5] mg/dl [p < 0.001]).
- No significant changes occurred in glycemic control for either group over 12 months.
- The 3-year overall survival was estimated at 72% (95% CI: 60–86%) for patients with DM versus 80% (95% CI: 70–92%) for patients without DM (p = 0.82).
